# High circadian stimulus lighting therapy for depression: Meta-analysis of clinical trials

**DOI:** 10.3389/fnins.2022.975576

**Published:** 2022-10-06

**Authors:** Li Zhou, Dandan Hou, Yang Wang, Sicong Zhou, Yandan Lin

**Affiliations:** ^1^Institute for Electric Light Sources, School of Information Science and Technology, Fudan University, Shanghai, China; ^2^Institute for Six-sector Economy, Fudan University, Shanghai, China; ^3^Intelligent Vision and Human Factor Engineering Center, Shanghai, China; ^4^Human Phenome Institute, Fudan University, Shanghai, China

**Keywords:** lighting therapy, circadian stimulus, depression, meta-analysis, non-visual effects

## Abstract

Although bright light therapy (BLT) has been widely used in the clinical treatment of depression, the antidepressant effect of BLT is not well understood. Considering the connection between depression and disrupted circadian rhythm, we assumed the model of human circadian phototransduction could be more accurate in evaluating the efficacy of BLT for depression compared to light level and spectrum. A systematic review and meta-analysis were conducted and the CS (circadian stimulus) model was used to quantify the efficacy of lighting in BLT. Articles published up to June 2022 were searched in COCHRANE, EMBASE, MEDLINE, and Web of Science. Randomized clinical trials included articles using high circadian stimulus (H-CS, CS > 0.1) as lighting therapy for people with depressive disorder vs. a control group (CS < 0.1). The treatment effect was estimated by calculating the mean difference (MD) with 95% confidence intervals (CIs). Seven trials involving 258 participants met the inclusion criteria. In this sample size, H-CS lighting was associated with a significant reduction in depressive symptoms (MD = −5.56, 95% CI = −9.22 to −1.90, *P* = 0.003, *I*^2^ = 64%). According to the meta-analysis, CS can be employed for the clinical evaluation of BLT for patients with depressive disorder and exposure to H-CS lighting significantly reduced depressive symptoms among adults. A range of CS > 0.57 was obtained, during which different lighting parameter combinations (e.g., light levels, spectra, duration, and light distribution) could achieve better treatment for depression.

**Systematic review registration:**

https://www.crd.york.ac.uk/prospero/display_record.php?ID=CRD42021253648.

## Introduction

BLT has been proven to be an effective and comparatively safe form of depression therapy. It is widely used in clinical practice, and is included in therapeutic guidelines for both seasonal affective disorder (SAD) and non-seasonal depression (NSD) (Lam and Levitt, 1999; Al-Karawi and Jubair, 2016). Electric lighting for 0.5–6 h/day (Lewy et al., 1986; Partonen, 1994) for a week or more has been shown to alleviate depressive symptoms for patients with depression diagnosed by standard clinical methods (Al-Karawi and Jubair, 2016). With advancements in basic science and electric lighting technology, it is now possible to quantify the dosing of lighting therapy. White light is currently used as the standard treatment spectrum for SAD and NSD (Al-Karawi and Jubair, 2016; Ravindran et al., 2016). In some studies, blue and green lights (Benedetti et al., 2003; Anderson et al., 2009) have also been shown to induce a positive therapeutic effect. The illumination is another mode of quantification, and cool-white sources with light level of 10,000 lx have been widely recommended in guidelines on treating depression (Golden et al., 2005). However, the Cochrane review of BLT for NSD concluded that there was no difference between the effects of bright light (>2500 lx) and the control groups (2500 lx maximum) (Kripke, 1998).

To explore the mechanism of light on human physiology, the non-visual effects should be distinguished from the visual effect. Visual effects come from the visual pathway, which extends from retinal photoreceptor cells to the visual cortex area (Kolb et al., 1995). The human eye perceives light in the visible spectrum with various photoreceptor cells, and different wavelengths contribute differently to the perception of vision. The visual perception is quantified by the photopic luminous efficiency function V(λ), which is the basic of photometric values such as illuminance (lux) (CIE, 2020). However, with the discovery of intrinsically photosensitive retinal ganglion cells (ipRGCs) and their non-visual effects such as mood changes (Berson et al., 2002; Lucas et al., 2003; Munch and Wirz-Justice, 2018), there is reason to believe that the light parameters used for characterizing vision may be inappropriate for evaluating the effects of non-vision neural pathways from the retina. Thus, although the parameters (e.g., lux, correlated color temperature) of the visual pathway are most commonly used to evaluate lighting, they may not be suitable to quantify the dose of lighting therapy.

Clinical studies have shown an association between circadian disruption and depression. Circadian rhythm disturbances are present in both SAD and NSD (Germain and Kupfer, 2008; McClung, 2011). Compared with normal people, patients with depression experience a delayed secretion of melatonin before bedtime (Bunney and Potkin, 2008). Melatonin is a pineal hormone regulated by the circadian clock, which is a reliable marker of the circadian rhythm (Peschke and Mühlbauer, 2010). It is worth noting that melatonin can be mediated by light exposure through the non-visual pathway (Berson et al., 2002). Therefore, the regulation of melatonin levels by lighting intervention may be a possible mechanism of BLT for both SAD and NSD.

In order to further validate the relationship between circadian regulation and BLT, the model of human circadian phototransduction is needed to quantify light as a stimulus for the circadian system. According to the non-visual physiological effects in humans, retinal photoreceptor cells respond to light, which then send the signals to the biological clock in the hypothalamic suprachiasmatic nucleus (SCN) (Wright and Lack, 2001). CIE 026 recommended using spectral sensitivity curves for five retinal opsin proteins (melanopsin, rhodopsin, S-, M-, and L-cone opsin), obtaining the equivalent illuminance corresponding to each opsin protein (CIE, 2018; Brown et al., 2022). Though ipRGCs are central to the non-visual pathway, ipRGCs also receive signals originating from rods and cones (Do, 2019). They are all involved in the circadian system phototransduction. Therefore, the equivalent illuminance corresponding to individual opsin proteins cannot completely represent the circadian response to light.

Another possible model is the circadian stimulus (CS), developed by Rea et al., which is based on the principles of retinal neurophysiology and psychophysics experiments (Rea and Figueiro, 2018; Rea et al., 2021a,b). CS takes into account the spectral sensitivity of the three photoreceptors (cones, rods, and ipRGCs) and can be used to characterize both the spectral sensitivity and the response magnitude characteristic of the circadian phototransduction circuit (Rea et al., 2021a). The latest model of CS is quantified in terms of spectrum, light levels, exposure time (duration of the circadian stimulus), and lighting distribution across the retina (Rea et al., 2021a). Melatonin suppression by the pineal gland at night was used as the outcome measure. Compared with single photoreceptor, the CS model can more accurately quantify circadian phototransduction. In the present study, CS was adopted as a new indicator to evaluate the therapeutic efficacy of lighting.

Here, we present a systematic review and meta-analysis of the CS model, uniting the multiple elements of lighting mentioned in the reviewed BLT studies. The Mean Difference (MD) of the depression scale was calculated to assess the efficacy of BLT. Using the reported light levels, spectral data, and the lighting distribution over time and space, the findings of the existing trials were screened based on the CS values. A systematic literature review and meta-analysis were conducted to verify the correlation between CS value and therapeutic effect, thus providing scientific metrics for lighting dose quantification of lighting therapy in clinical applications.

## Methods

### Literature search

This systematic review was registered with PROSPERO under code number CRD42021253648 and was conducted in accordance with the PRISMA guidelines (Moher et al., 2009). Articles published before 2021 were searched in the Cochrane Library, EMBASE, MEDLINE, and Web of Science. Various synonyms from MeSH (“depressive disorder,” “phototherapy,” and “randomized controlled trial”) were entered in these electronic databases to improve the sensitivity of the search. Supplementary searches of relevant systematic reviews and references within the included articles were performed manually.

### Selection of parameters for describing the circadian stimulus

As mentioned previously, CS refers to the spectral sensitivity and the response magnitude characteristic of the circadian phototransduction circuit, and melatonin suppression is used as the outcome measure. The specific calculation process for CS was based on the studies of Rea et al. (2021a, 2022). The calculation formula is as follows (Eq. 1):


$$\begin{array}{l} CS_{t,\;f}=0.7\;*\;\left[1-\frac{1}{1+{\left(\frac{t\;*\;f\;*\;CL_{A}}{355.7}\right)}^{1.1026}}\right] \end{array}$$

where *t* represents the exposure durations of circadian light exposure, *f* represents the spatial distribution of circadian light exposure (Rea et al., 2021a), *CL*_*A*_ represents circadian light illumination determined by the physiological properties of human eyes and stimulus light source (spectrum and level) (Rea et al., 2022). The calculation of CS requires the spectral power distribution. However, some of the previous related studies did not report detailed spectral information. Therefore, the CIE standard light source closest to the description of the light conditions given in these studies was used as a substitute source of spectral information for the CS calculation. The specific substituted spectral information is shown in Table 1.

**Table 1:** Characteristics of the included trials.

**Trial**	**Country**	**Sample**	**Intervention**	**Control**	**SPD for CS calculation**	**Duration**	**Mood outcomes**	**The other outcomes**	**Results**
Desan et al. (2007)	USA	23 participants with SAD aged 18 to 65 y who met DSM-IV criteria	1,350 lx CS = 0.57	No lighting CS <0.01	The Lifebook Company Ltd., Alberta, Canada	30 min/day before 8 am for 4 weeks	-SIGH-SAD	-Mean times of sleep	The SIGH-SAD scores of active treatment were significantly lower than control group (*p* = 0.027). There were no significant changes in time of sleep in either group.
Glickman et al. (2006)	USA	24 patients (19 females, 5 males) with major depression with a seasonal pattern who met DSM-IV criteria	10,000 lx CS = 0.68	23 lx CS = 0.02	CIE F2*	45 min/day in the morning for 3 weeks	-SIGH-SAD	-Sleep-wake time	The short-wavelength light treatment decreased SIGH-SAD scores significantly more than the dimmer red-light condition (*F* = 6.45, *p* = 0.019 for average over the post-treatment times).
Goel et al. (2005)	USA	20 patients with major depressive disorder who met DSM-IV criteria	10,000 lx, 3,000°K CS = 0.67	No lighting CS <0.01	CIE F4*	60 min/day upon awakening for 5 weeks	-SIGH-SAD	-Circadian phase -Sleep time	SIGH-SAD score improvement was 53.7% for bright light and 17% for low-density ions. Remission rates (pre- to-post-treatment reduction in SIGH-SAD score to ≤ 8) were 50%, and 0%, respectively. There were no significant changes of sleep and melatonin in either group.
Koorengevel et al. (2001)	Netherlands	29 patients (21 females, 8 males) showing recurrent major depressive disorder with a seasonal pattern who met DSM-IV criteria	13,000 lx CS = 0.69	0 lx CS <0.01	Ohmeda Biliblanket Plus Phototherapy System 455–540 nm	8:00–11:00 am for 2 weeks	-SIGH-SAD−21-items HRSD−8 items ATYP	-DLMO	Between conditions, no significant differences were observed in clinical scores, the self-ratings on mood and alertness, and in timing of the DLMO before and directly after treatment.
Lieverse et al. (2011)	USA	89 outpatients (58 females, 31 males) with MDD aged ≥60 y meeting the criteria in the 15-item version of the GDS	7,500 lx, mist-blue filter CS = 0.69	50 lx, blood-red filter CS <0.01	Philips Bright Light Energy HF 3304; Koninklijke Philips Electronics NV, Eindhoven, the Netherlands	60 min in the morning for 3 weeks	-SIGH-SAD−6 items HAM-D−8 items ATYP -MADRS	-Sleep time -Cortisol levels -Melatonin levels	HAM-D scores improved more with BLT than with placebo (21%; 7%−31%; *p* = 0.001) and the get-up time was advanced by 3% (*p* = 0.001) and the 24-h urinary free cortisol level was 37% lower (*p* = 0.003) compared with the placebo group. The evening salivary cortisol level had decreased by 34% in
									the BLT group compared with an increase of 7% in the placebo group (*p* = 0.02).
Rastad et al. (2008)	Sweden	48 adults (20–68 years) with SAD or S-SAD who met DSM-IV	4,300 lx, full-spectrum CS = 0.67	No lighting CS <0.01	D65*	1.5–2 h/day (6:00–9:00 am) for 3 weeks	- SIGH-SAD−21 items HAM-D−8 items ATYP	/	A significant main effect was observed for the light room therapy group. Fifty-four percent (*n* = 13/24) showed ≥50% improvement while no improvement was seen in the control condition (*n* = 0/24).
Strong et al. (2009)	USA	25 patients aged 44.3 ± 12.6 y with SAD who met DSM-IV criteria	Blue: 176 lx, 420–520 nm, peak ≈ 470 nm CS = 0.57	Red: 201 lx, 600–670 nm, peak ≈ 635 nm CS <0.01	LED, 470 nm LED, 635 nm	45 min/day (6:00–8:00 am) for 3 weeks	-SIGH-SAD -HAMD-17	/	HAMD-17 scores improved more under the blue-light condition (51%) than under the red-light condition (32%) (*p* = 0.05). Patients in both conditions had prominent anxiety (HAMD subscale score, mean = 5.6, SD = 2.01), reduced sleep quality (PSQI score, mean = 8.3, SD = 3.4).

SPD, spectral power distribution; CS, circadian stimulus; DSM-IV, diagnostic and statistical manual of mental disorders, 4th edition; SAD, seasonal affective disorder; SIGH-SAD, structured interview guide for the Hamilton depression rating scale, seasonal affective disorders; S-SAD, sub-clinical SAD; MDD, major depressive disorder; GDS, geriatric depression scale; HAM-D, Hamilton depression rating scale; HRSD, Hamilton rating scale of depression; ATYP, Atypical symptoms; DLMO, dim light melatonin onset; MADRS, Montgomery-Asberg depression rating scale; MRS, Mania rating scale; SD, standard deviation.

^*^Standard CIE light source.

Advantageously, CS = 0.1 is established as a clear and quantifiable cutoff level that has demonstrated no effect on the circadian rhythm (Alzahrani et al., 2022). Therefore, CS = 0.1 was set as the demarcation criteria for the intervention group and the control group of the light therapy, CS < 0.1 was defined as the low CS group (the control group), and CS > 0.1 as the high CS group (the intervention group).

### Inclusion criteria

**Figure 1:**
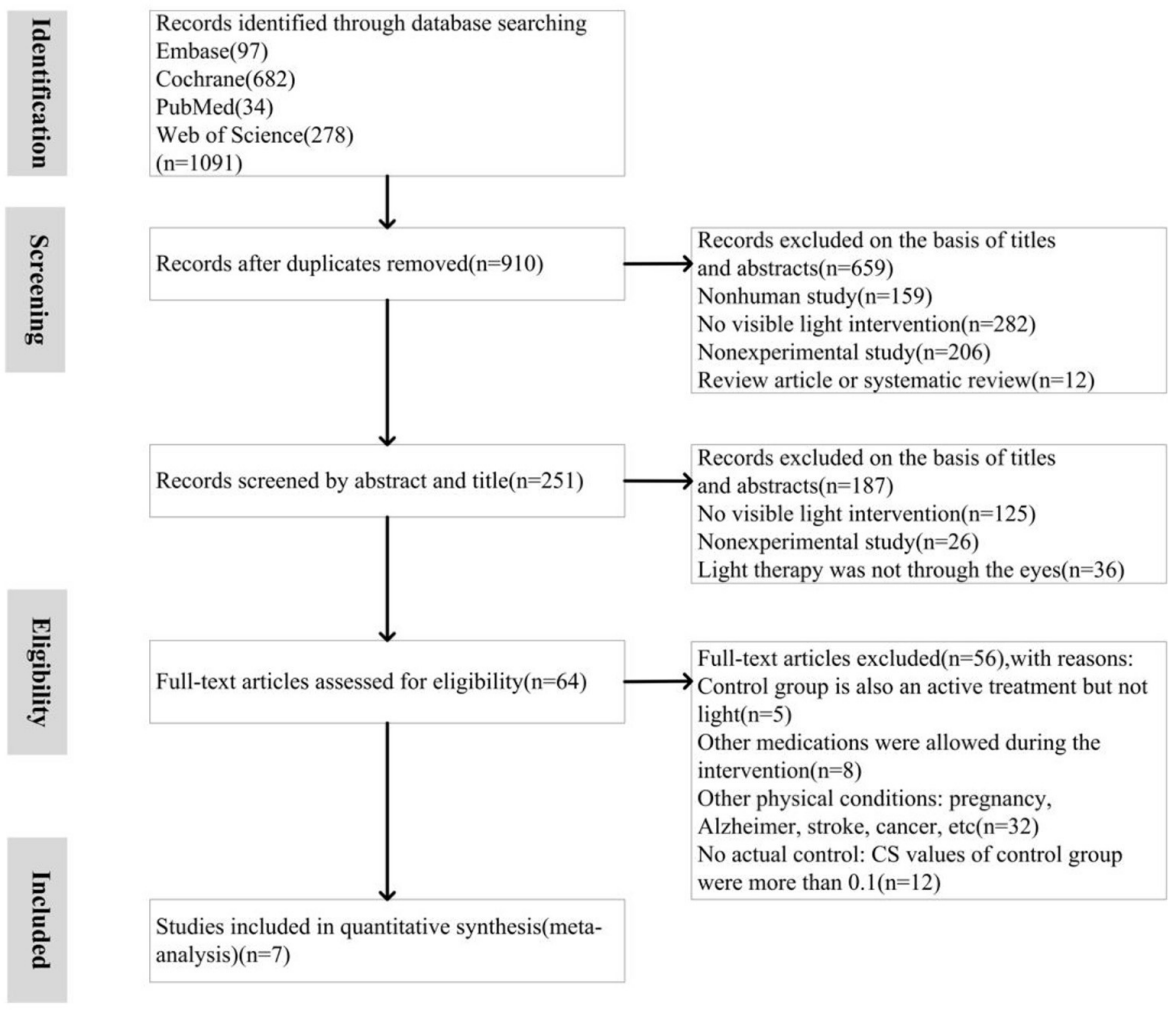
Meta-analysis of high circadian stimulus (H-CS) lighting therapy for depression: Trial selection process.

Studies that met the following criteria were included: (A) clinical trials with randomized allocation; (B) patients diagnosed with SAD or NSD by standard clinical methods; (C) patients not in a specific physiological state (such as the period before and after pregnancy) and having no other physiological diseases; (D) patient consented to light therapy as the only independent intervention, with no antidepressant medication or other treatment modalities; (E) the CS of the control/placebo groups (e.g., treated with dim lighting or negative air ions) was relatively low (< 0.1) while the CS of the intervention group was relatively high (>0.1); (F) the outcome variable and effect size were reported in terms of standardized depression scales. The inclusion and exclusion protocols are summarized as a PRISMA flow chart in Figure 1.

### Data extraction

The following data were extracted from the identified trials: participant characteristics, sample size, assessment modalities, types of depression, sex, diagnostic criteria for depression, illumination parameters, duration of lighting intervention, the definition of the control group, and basic conclusion. The parameters of the interventional lighting used to calculate CS included illuminance, spectra, duration of exposure, and light distribution (illumination configuration).

### Study quality assessment

Two reviewers independently assessed trial quality using the “Risk of Bias Table” from the Cochrane Handbook. This table was amended to include selection bias, performance bias, detection bias, attrition bias, and reporting bias, and was used to evaluate the randomization and blinding methods, outcome measures, and loss to follow-up of the included trials. The final score for each study was quantified as “Low risk,” “Unclear risk,” or “High risk.”

### Data analysis

Although the identified trials included cases of seasonal and non-seasonal depression, they used the same continuous outcome variable, namely SIGH-SAD scores. The mean difference (MD) and weighted mean difference with 95% CI were chosen as the effect size. The meta-analysis factored in the mean, standard deviation (SD), sample size, and weight of each original study. In this study, the weight was the inverse of the variance calculated by Review Manager Software (version 5.3.0). The heterogeneity was assessed by evaluating the *P* and *I*^2^ values, and different effect models were selected according to the results of the heterogeneity analysis. In addition, sensitivity analysis was performed to test the stability of the model, which involved examining the heterogeneity and effect size before and after a specific study was deleted.

## Result

### Study characteristics

Of 1,091 studies identified in the database, 7 were included in the meta-analysis. Figure 1 depicts the selection process. Notably, all identified studies had been included in previously published reviews. However, based on the inclusion criteria, we studied both seasonal and non-seasonal depression and intervention/control conditions after parameter conversion (CS value of the intervention group > 0.1; CS value of the control group < 0.1). The lighting parameters were extracted from the 7 clinical trials and analyzed. The results for a total of 258 participants were evaluated, of which 130 patients received H-CS lighting therapy (CS > 0.1) and 128 patients received a placebo control H-CS lighting therapy (CS < 0.1). All patients were diagnosed with standardized screening measures: seven datasets were based on clinical diagnosis using DSM-IV, and one used the Geriatric Depression Scale for elderly participants. All the included trials quantified improvements in depression using standardized scales.

### Risk-of-bias assessment

**Figure 2:**
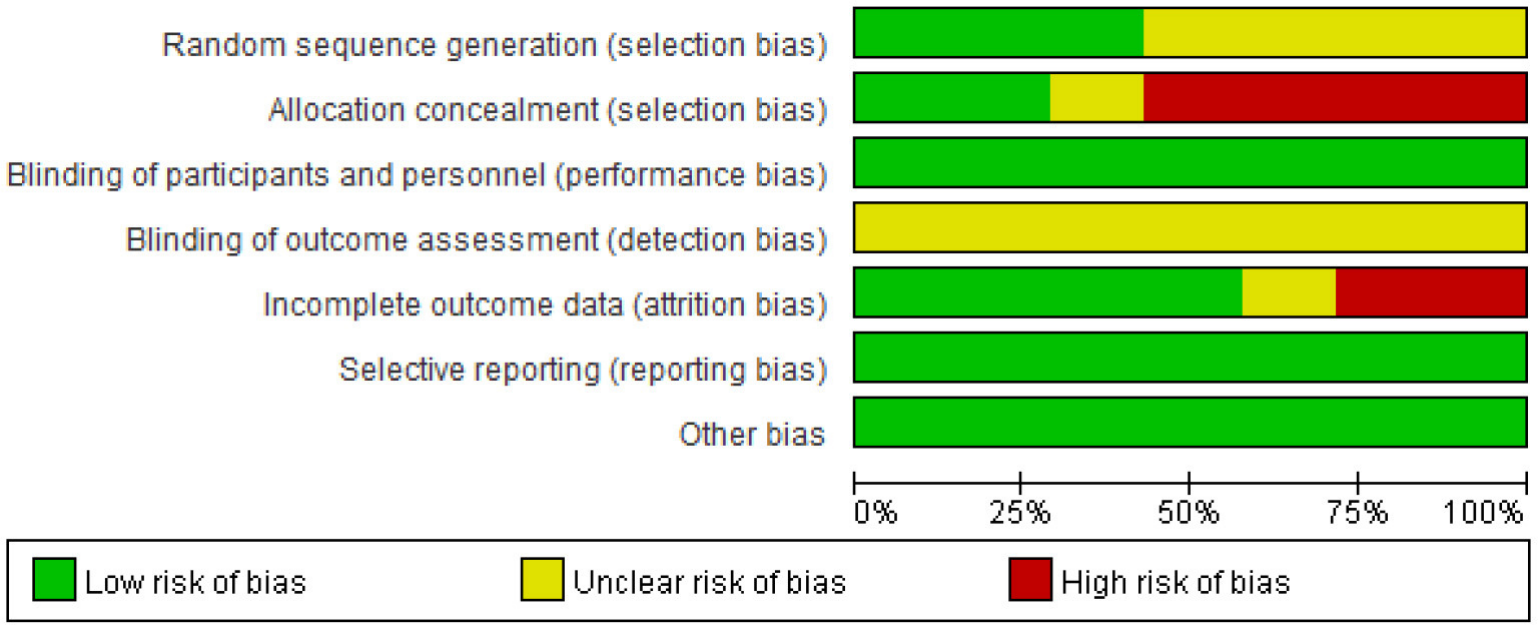
Risk-of-bias graph: Shown are the judgments about each risk-of-bias item, presented as percentages of all included studies.

The eligible studies were all randomized controlled trials and the quality of evidence was evaluated with the Revised Cochrane Risk of Bias Tool (Evans et al., 2015), as displayed in Figure 2. Among these 7 studies, two studies adopted a random number table (Goel et al., 2005; Lieverse et al., 2011) and one study used a computer-generated randomization sequence (Rastad et al., 2008) to generate the groups. Since the four remaining studies did not specify any random sequence generation methods, they were categorized as a moderate risk of selection bias. Two studies reported allocation concealment using opaque envelopes (Lieverse et al., 2011) and specific examiners (Koorengevel et al., 2001). The five remaining studies were at high risk of bias in terms of allocation concealment because methods of allocation concealment were not mentioned. Two studies were at high risk of attrition bias as participants withdrew from the experiment (Glickman et al., 2006; Desan et al., 2007).

Notably, lighting interventions are distinct from pharmaceutical interventions as lighting properties are intuitively recognized by the human eye. Thus, while the participants may not recognize experimental drugs and vitamins, they can easily distinguish different colors and light levels. Participants were told that the importance of color and different wavelengths remained uncertain (Strong et al., 2009). Thus, the blinding of outcomes and personnel was unclear.

### Clinical efficacy of H-CS lighting

#### Main analysis

**Figure 3:**
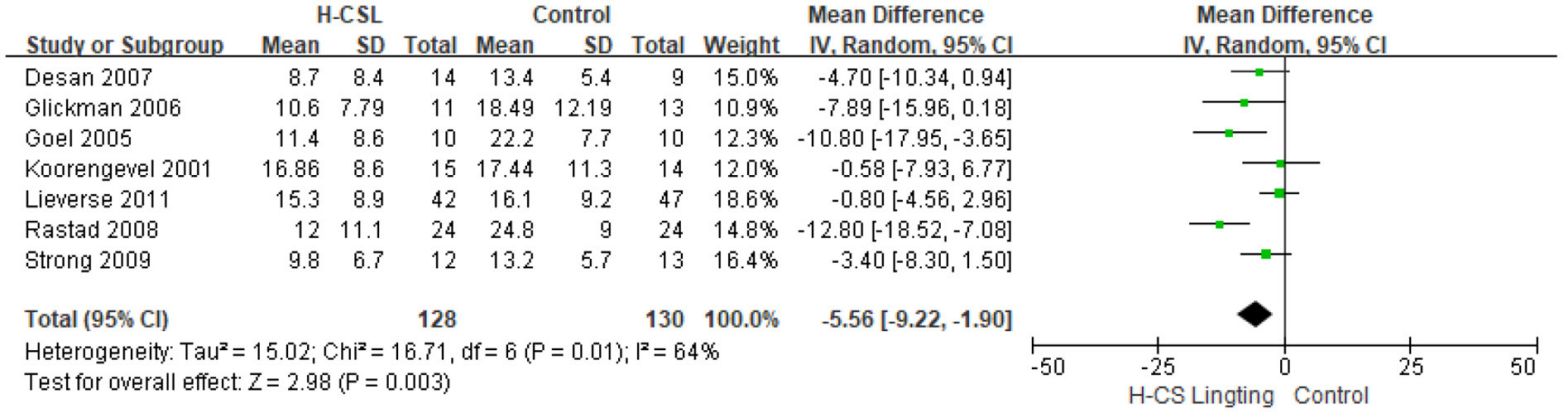
Forest plot comparison: High circadian stimulus lighting (H-CS) group vs. the control group. Outcome: Mean difference.

Table 1 presents a synopsis of the included studies, briefly describing the study design, sample, interventions, circadian stimulus values, outcomes, and results. The studies included a variety of measures, methods, samples, and analytical approaches. The meta-analysis included all 7 eligible estimates addressing SAD, NSD (Goel et al., 2005) and geriatric depression (Lieverse et al., 2011). The meta-analysis revealed that H-CS lighting therapy was associated with a significant reduction in depressive symptoms (MD = −5.56, 95% CI = −9.22 to −1.90, *P* = 0.003, *I*^2^ = 64%) (Figure 3). This finding implies that the H-CS lighting intervention showed a significant reduction of depressive symptoms compared to the corresponding control group (CS < 0.1).

#### Sensitivity analysis and subgroup analysis

**Figure 4:**
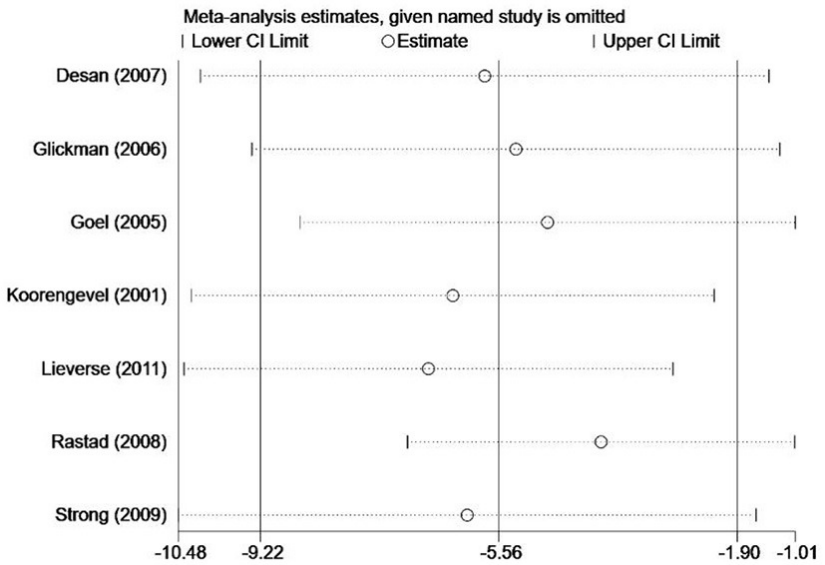
Sensitivity analysis of meta-analysis.

Sensitivity analysis was conducted to assess the stability of the results and the influence of each study on the total estimate, by omitting one study at a time (Figure 4). When any single finding is excluded, sensitivity analyses reveal that the overall result does not change. Since only one paper evaluated geriatric depression (i.e., depression in participants over 60 y of age), subgroup analysis was performed to assess the influence of age on the association between light therapy and depression. As displayed in Table 2, the results for the older adults subgroup (Lieverse et al., 2011) were not significant (MD = −0.8, 95% CI = −4.56 to 2.96). Several other studies (Rastad et al., 2008; Strong et al., 2009) included older adults and non-elderly participants. The studies that provided an accurate age range of the non-elderly participants (Koorengevel et al., 2001; Goel et al., 2005; Glickman et al., 2006; Desan et al., 2007) were included in the non-elderly subgroup (MD = −5.85, 95% CI = −9.97 to −1.73, *P* = 0.005, *I*^2^ = 29%). Data were also subgrouped based on the length of treatment. The result showed that the short-term exposure to H-CS lighting therapy (MD = −4.90, 95% CI = −9.57 to −0.23, *P* = 0.04, *I*^2^ = 71%) was not significantly different compared to exposure to long-term exposure (MD = −7.34, 95% CI = −13.26 to −1.41, *P* = 0.02, *I*^2^ = 42%).

**Table 2:** Subgroup analysis of the efficacy of H-CS light therapy on depressive symptoms.

**Subgroup**	***N***	**Sample**	**MD**	**95% CI**	***P***	**Heterogeneity test (*I*^2^)**
Age
Non-elderly (< 60 y)	4	96	−5.85	[−9.97, –1.73]	0.005	29%
Older adults (>60 y)	1	89	−0.80	[–4.56, 2.96]	-	-
Duration of intervention
Short-term (1–3 weeks)	5	215	−4.90	[−9.57, −0.23]	0.04	71%
Long-term (≥4 weeks)	2	43	−7.34	[−13.26, −1.41]	0.02	42%

H-CS, high circadian stimulus; MD, mean difference; CI, confidence interval.

## Discussion

### Summary and interpretation of findings

CS can be used as a new metric quantification method of lighting, which might more accurately represent the dose of light therapy in depression than vision parameters. In this review, H-CS lighting was associated with a significant reduction in depressive symptoms across 7 studies regardless of participant characteristics (e.g., sex, SAD vs. NSD) or individual lighting features (e.g., light levels, spectra, duration, and light distribution).

The funnel plot does not reliably represent the risk of publication bias due to the small number of trials included in this study. Moreover, the presence of low sample size trials can indirectly contribute to publication bias (Sterne et al., 2000). However, eligible articles were screened to meet the requirements of the experimental and control groups based on the range of CS values reducing publication bias. The relationship between CS and therapeutic outcomes could not be obtained from the original articles because the CS measure was introduced only in the present study.

Some previous studies have already proposed that illuminance measurement in lux (lx) may be inadequate for evaluating the dose in BLT studies. For example, the therapeutic effect of blue-appearing light with an illumination of 98 lx was similar to that of white-appearing light with an illumination of 700 lx (Anderson et al., 2009). Notably, the CS values of both groups were equal (0.6) in the research. Several studies (Sumaya et al., 2001; Anderson et al., 2009; Meesters et al., 2018) were not included in our analysis due to the CS values being similar between groups. All of these studies reported decreased depression ratings in both the intervention and control groups, with no statistically significant differences.

Furthermore, another experiment by Anderson et al. showed that both the blue light-free group (CS = 0.30 ± 0.03) and the blue light group (CS = 0.68 ± 0.06) had a significant treatment effect and there was no significantly different between the two groups (Anderson et al., 2016). The results of Anderson's study suggested that depression can be effectively treated when CS > 0.3, however, as the CS values of both groups in this study were greater than 0.1, this research was not included in the present study. According to the meta-analysis result, a certain range of CS values (0.57–0.7) exerts a positive effect on depression but is not restricted in high illumination or specific wavelengths.

In the subgroup analysis based on age, non-elderly patients showed a statistically significant effect in response to H-CS lighting therapy. Only one article specifically investigated the elderly population and showed no significant effect when treated with H-CS lighting. Similarly, a reduction in responsiveness to photic stimuli in the circadian timing system in older adults was reported (Kim et al., 2014). This difference may be attributed to the physiology of the elderly eye. With age, the retina, cornea, lens, and other structures in the visual organs undergo substantial changes, including a reduction of lens transparency and changes in the crystalline lens color, which can result in variations in the refractive index of the lens. These age-related lens changes increase short wavelength light absorption, impairing the ability to absorb short wavelengths light (Mellerio, 1987). Another possible contributing factor is that, as described in previous studies, melanopsin is a short-wavelength-sensitive opsin located in ipRGCs that plays a key role in the non-vision pathways (Berson et al., 2002). Hence, elderly patients receive markedly weaker circadian stimulation than non-elderly patients, prompting caution in the evaluation and quantification of CS in elderly patients. Another study also observed the positive effects of lighting with CS = 0.3 on depression therapy in older persons at risk for Alzheimer's disease (Figueiro and Kales, 2021). Therefore, more clinical research focusing on elderly patients is required due to the limited number of studies.

Further subgroup analysis was performed based on the duration of the intervention. Analyses of two subgroups showed consistent results, with the long-term group (≥4 weeks) showing no significant advantage over the short-term group (1–3 weeks). Thus, the non-visual impact of light may have a cumulative efficacy over a limited period and reaches saturation after a specific duration.

### Limitations

Nevertheless, this study has several limitations. Only seven studies (*N* = 258) were included in the meta-analysis, which restricts the quality of the evidence. Notably, according to the inclusion criterion (E) for the intervention group, when the CS value was larger than 0.1, the research can be incorporated into the meta-analysis. However, the article screening results yielded only CS values above 0.57 in the intervention group, and intermediate CS values were missing. Therefore, the treatment effect of CS in depression could only be determined for two extreme cases. In conclusion, a significant treatment effect of H-CS photomedicine was achieved with CS higher than 0.57; the present analysis did not establish a therapeutic effect for CS in the range of 0.1–0.57.

Another issue that requires attention is the heterogeneity of the populations in this study. The included sample consisted of SAD and NSD, and also cover patients of different ages and depression severity. Due to the physiological peculiarities of depressed patients, H-CS lighting therapy requires further validation. In the future, more targeted phototherapy studies are necessary to help discovering the specificity of phototherapy for different natures of depression (type, gender, age, degree of illness, etc.). Future trials should also include additional intermediate CS values to establish a model based on treatment outcomes and non-visual light parameters. Moreover, in comparison with visual parameters, non-visual optical parameters have been poorly researched to date. Future studies may identify new parameters that are more appropriate for the accurate evaluation of the therapeutic effect of lighting.

### Clinical implications

By controlling the spectrum and level of the light for depression disorder therapy, H-CS light mode may be achieved with a preferred color or a gentle light level, improving comfort for the patients, and increasing the effects of light therapy.

## Conclusion

Data from this review support the use of CS as a new quantitative measure for the evaluation of bright light therapy in depression. Compared with visual parameters, CS accounts for spectra, illumination, duration, and spatial distribution and how the circadian system responds to them. Based on this sample size, a relatively better therapeutic effect was achieved when the CS value was above 0.57 (H-CS lighting therapy).

Furthermore, additional clinical research should be conducted by using the same CS values to validate this model. Meanwhile, the therapeutic effect of light therapy with CS in the 0.1–0.57 range remains unknown, and future studies should investigate the effect of these intermediate CS values.

## Data availability statement

The original contributions presented in the study are included in the article/supplementary material, further inquiries can be directed to the corresponding author.

## Author contributions

YL supervised the research and built the article framework. All authors contributed to the literature search. Data collection and analysis were performed by LZ and DH. Study quality and risk-of-bias assessments were performed by LZ, YW, and SZ. The first draft of the manuscript was written by LZ and all authors commented on previous versions of the manuscript. All authors have read and approved the final manuscript.

## Funding

This study was funded by the Shanghai Municipal Science and Technology Major Project (Grant No. 2017SHZDZX01).

## Conflict of interest

The authors declare that the research was conducted in the absence of any commercial or financial relationships that could be construed as a potential conflict of interest.

## Publisher's note

All claims expressed in this article are solely those of the authors and do not necessarily represent those of their affiliated organizations, or those of the publisher, the editors and the reviewers. Any product that may be evaluated in this article, or claim that may be made by its manufacturer, is not guaranteed or endorsed by the publisher.
